# Integrated Solid/Nanoporous Copper/Oxide Hybrid Bulk Electrodes for High-performance Lithium-Ion Batteries

**DOI:** 10.1038/srep02878

**Published:** 2013-10-07

**Authors:** Chao Hou, Xing-You Lang, Gao-Feng Han, Ying-Qi Li, Lei Zhao, Zi Wen, Yong-Fu Zhu, Ming Zhao, Jian-Chen Li, Jian-She Lian, Qing Jiang

**Affiliations:** 1 Key Laboratory of Automobile Materials (Jilin University), Ministry of Education, and School of Materials Science and Engineering, Jilin University, Changchun 130022, China; 2 These authors contributed equally to this work.

## Abstract

Nanoarchitectured electroactive materials can boost rates of Li insertion/extraction, showing genuine potential to increase power output of Li-ion batteries. However, electrodes assembled with low-dimensional nanostructured transition metal oxides by conventional approach suffer from dramatic reductions in energy capacities owing to sluggish ion and electron transport kinetics. Here we report that flexible bulk electrodes, made of three-dimensional bicontinuous nanoporous Cu/MnO_2_ hybrid and seamlessly integrated with Cu solid current collector, substantially optimizes Li storage behavior of the constituent MnO_2_. As a result of the unique integration of solid/nanoporous hybrid architecture that simultaneously enhances the electron transport of MnO_2_, facilitates fast ion diffusion and accommodates large volume changes on Li insertion/extraction of MnO_2_, the supported MnO_2_ exhibits a stable capacity of as high as ~1100 mA h g^−1^ for 1000 cycles, and ultrahigh charge/discharge rates. It makes the environmentally friendly and low-cost electrode as a promising anode for high-performance Li-ion battery applications.

Growing demands for high-energy and -power storage/delivery technology in many important applications^1^,^2^,^3^,^4^,^5^,^6^, including hybrid vehicles, portable electronic equipments and renewable energy, have stimulated intensive research on energy storage devices with the capability of rapid charge/discharge rates^1^,^2^,^4^,^7^,^8^,^9^,^10^,^11^,^12^,^13^,^14^. A major challenge in the realization of battery-like capacity and supercapacitor-like rate performance in an electrochemical energy storage system is to develop electrode materials that can combine the advantages in both devices^12^,^15^. Capacitive charge storage enables high-power capability in supercapacitors by using nonFaradic surface ion adsorption (double-layer capacitance) and Faradic surface redox reaction (pseudocapacitance)^11^,^15^,^16^,^17^ whereas in Li-ion batteries Faradic insertion/extraction of Li^+^ ions occurs in the bulk of the electroactive materials^2^,^15^,^18^. As a consequence, Li-ion batteries assembled with the conventional electrodes composed of millimeter-sized powders exhibit high-energy storage, but are subjected to low recharging rates because of kinetic problems of ion and electron transports, as well as poor cycling stability owing to large volume changes during charge/discharge^1^,^2^,^15^,^18^. Approaches to circumvent these limitations of Li-ion batteries include developing new electroactive materials with alternative electrochemical reactions^1^,^7^,^19^,^20^ and designing multifunctional composite electrodes^21^ for an aim at minimizing primary resistances in charge/discharge processes owing to ion and electron transports in electrodes and current collectors, as well as electrochemical reactions in electrodes^4^,^22^. Transition metal oxides (M_x_O_y_, where M denotes Co, Ni, Cu, Fe or Mn) as anode materials offer alternative opportunity to further improve the performance of Li-ion batteries because of the storage of high-capacity charge (~1000 mA h g^−1^) *via* redox or conversion reactions occurring at both surface (capacitive storage)^8^,^11^,^17^ and interior (Li insertion/extraction)^1^,^7^,^8^,^9^,^18^,^19^,^23^,^24^. The complete electrochemical reduction of metal oxides in light of the conversion reaction, 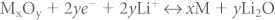, produces a composite material consisting of metallic nanoclusters dispersed in an Li_2_O matrix, displaying high reversibility^1^,^19^,^23^. Since the original work of Poizot *et al.*^7^, nanomaterials of metal oxides, such as MnO_2_^25^,^26^,^27^,^28^ and copper oxides (Cu_2_O and CuO)^29^,^30^,^31^,^32^,^33^,^34^,^35^,^36^, have been intensively studied as anode materials for Li-ion batteries due to their high abundance, low cost and environmental benignity, demonstrating the strong dependence of electrochemical performances on particle size and morphology of precursors^7^,^19^,^25^,^26^,^27^,^28^,^29^,^30^,^31^,^32^,^33^,^34^. Although there is an optimum precursor for each metal oxide system to show good capacity retention^7^,^19^,^34^, their poor electronic conductivity (for example, MnO_2_ and CuO in ~10^−5^–10^−6^ and ~10^−2^ S cm^−1^, respectively)^17^,^37^, large volume expansion and low energy efficiency during repeated lithium cycling processes limits the potential applications in practical Li-ion batteries^1^,^19^,^23^,^25^,^26^,^27^,^28^,^29^,^30^,^31^,^32^,^33^,^34^,^35^,^36^. To improve their electron transport and cycling performance, there have been initial explorations on developing composite electrodes by employing conductive agents, such as metal pillars^23^ or substrates^35^, carbon nanotubes (CNTs)^8^,^10^,^38^ and nanohorns^39^, graphene^9^,^40^,^41^, to serve as conductive pathways of metal oxides. Nevertheless, the electrodes, which are assembled by a traditional approach to mix these low-dimensional active nanocomposites using polymeric binders, exhibit undesirably low electrical conductivity and ion transport owing to exceptionally low electron conductivity in the nanomaterials as well as the high contact resistances within nanomaterials and between the current collector and electrodes, essentially impeding their wide use in practical high-power Li-ion batteries^7^,^8^,^9^,^19^,^23^,^42^. Moreover, large volume changes during Li insertion/extraction lead to low power capability, severe capacity fading, and even electrode failure as a result of pulverization, aggregation and loss of electrical contact^43^.

Here, we report seamlessly integrated solid/nanoporous (S/NP) Cu/MnO_2_ hybrid bulk Li-ion battery electrodes, of which the three-dimensional (3D) NP Cu/MnO_2_ layer with bicontinuous nanopores/ligaments and intimate Cu/MnO_2_ interface enhances ion and electron transports while the charge storage of MnO_2_ is facilitated by a dual mechanism of capacitive and Li insertion/extraction processes. As a result of concurrent realization of minimizing primary resistances, producing stable Cu/MnO_2_ interface and accommodating large volume changes during charge/discharge, the constituent MnO_2_ delivers high energy at ultrahigh rates with outstanding cyclability.

## Results

**Figure 1:**
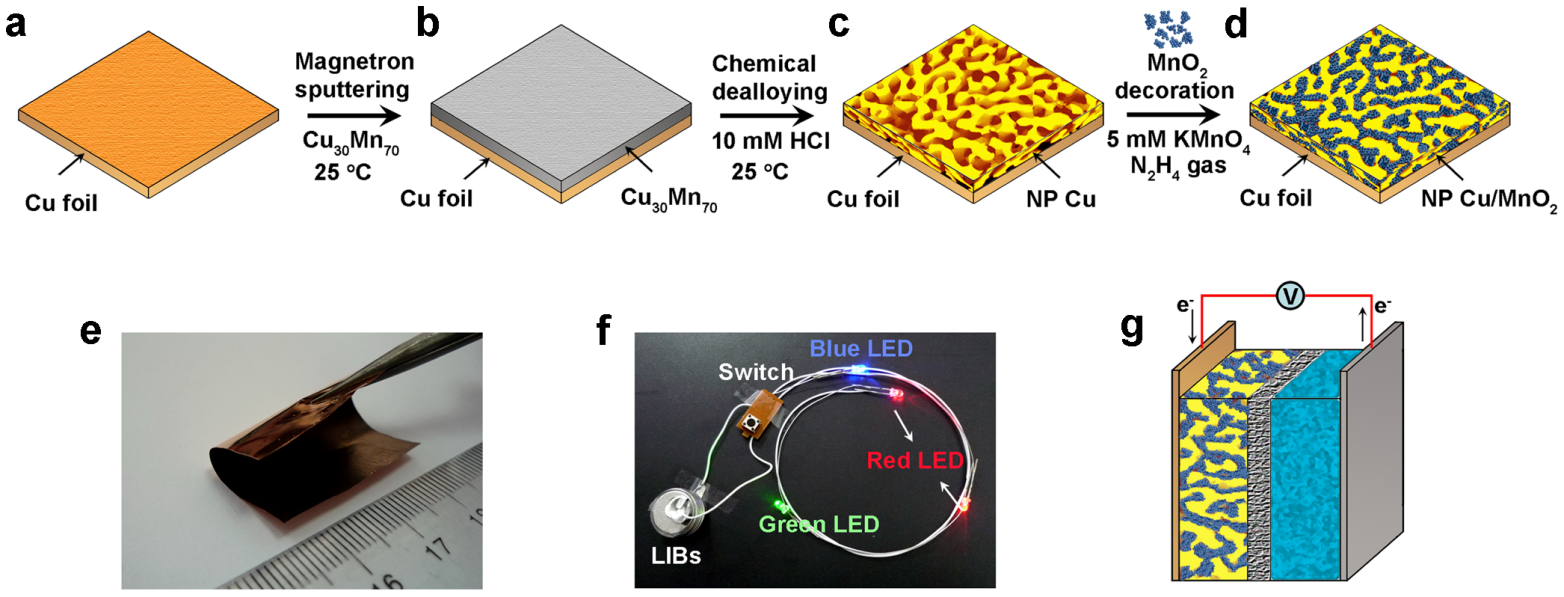
Hybrid bulk electrodes with 3D nanoporosity for Li-ion batteries. Scheme showing the fabrication of seamlessly integrated S/NP Cu/MnO_2_ bulk electrode: (a), Cleaned copper foil substrate; (b), Cu_30_Mn_70_ alloy film deposited on the copper foil by magnetron sputtering; (c), Nanoporous Cu layer on the copper foil produced by chemically dealloying Cu_30_Mn_70_ in diluted HCl solution; (d), Nanocrystalline MnO_2_ directly grown onto S/NP Cu skeleton using electroless plating. (e), Photograph of a flexible S/NP Cu/MnO_2_ hybrid bulk electrode being bend (2 × 3 cm^2^). (f), Four batteries assembled with S/NP Cu/MnO_2_ bulk electrodes power blue, red and green LEDs. (g), Schematic battery constructed with S/NP Cu/MnO_2_ and lithium foil as electrodes, 1 M LiPF_6_ in EC/EMC/DMC as electrolyte, and porous polymer as separator.

**Figure 2:**
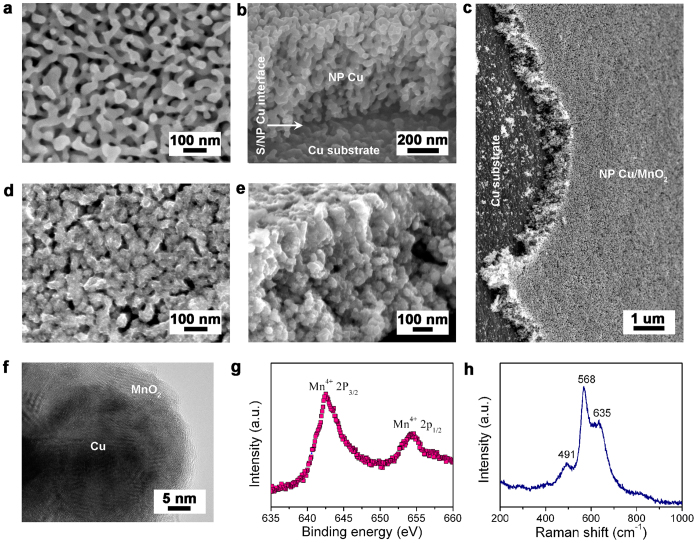
Microstructure characterization. (a), (b), Top-view and cross-sectional SEM images of S/NP copper with a characteristic length of ~50 nm. (c), (d), (e), Low-magnification, top-view and cross-sectional SEM micrographs of S/NP Cu/MnO_2_ after electroless plating. (f), Bright-field HRTEM image of the S/NP Cu/MnO_2_ hybrid. The hybrid nanostructure can be identified by the contrast between the bright MnO_2_ filler and the dark copper ligament. (g), XPS spectrum of Mn-*2p* orbit for the MnO_2_ incorporated into S/NP Cu sheet. (h), Raman spectrum of S/NP Cu/MnO_2_ hybrid bulk electrode.

The S/NP Cu/MnO_2_ electrodes are fabricated by a facile procedure, which involves synthesis of seamlessly integrated S/NP Cu skeletons and electroless plating of MnO_2_ into the nanoporous layer (Figs. 1a–e and Supplementary Fig. S1)^17^, for the use in coil cells (Figs. 1f, g). Following the deposition of Cu_30_Mn_70_ alloy films onto Cu foils via magnetron sputtering (Supplementary Fig. S2a), S/NP Cu skeletons are produced by chemical dealloying in the N_2_-bubbled HCl solution, during which less noble Mn is selectively dissolved while remained Cu forms nanoporous structure (Supplementary Fig. S2b)^44^,^45^,^46^. Figure 2a shows typical top-view scan electron microscope (SEM) image of as-dealloyed S/NP copper, demonstrating that a 800-nm-thick 3D bicontinuous nanoporous layer consisting of quasi-periodic Cu ligaments and nanopore channels with a pore size of ~50 nm (Fig. 2a and Supplementary Fig. S3a)^47^,^48^ is seamlessly jointed with Cu foil (Fig. 2b, Supplementary Fig. S4). Elemental mapping for Cu, Mn and O in the as-dealloyed NP Cu layer (Supplementary Fig. S5) reveals the negligible Mn (~1.14 wt.%) and O (~0.25 wt.%) remains that cannot be identified in high-resolution transmission electron microscope (HRTEM) image of Cu ligaments (Supplementary Fig. S6) and X-ray diffraction pattern (Supplementary Fig. S7). After electroless plating, MnO_2_ nanoparticles with the amount of ~12 wt.% are uniformly incorporated into pore channels of the entire NP Cu layer without any defect (Fig. 2e, Supplementary Figs. S2c and S8) while the hybrid electrodes maintaining open nanoporosity with the pore size of ~15 nm (Figs. 2c, d, Supplementary Fig. S3b). The loading of rough MnO_2_ nanocrystals with diameter of ~5 nm gives rise to a larger real surface area of S/NP Cu/MnO_2_ than that of bare S/NP Cu (Supplementary Figs. S3c, S8). HRTEM micrograph shows that well-crystallized MnO_2_ directly grows along the Cu ligaments with end-bonded contact (Fig. 2f)^49^, offering excellent electrical conductivity and stable interface between Cu and MnO_2_^17^,^49^. The chemical state of MnO_2_ is verified by X-ray photoelectron spectroscopy (XPS) survey, wherein Mn-2*p* core level spectrum displays two peaks at the binding energies of 642.5 eV and 654.4 eV, corresponding to Mn-2*p*_3/2_ and 2*p*_1/2_ orbits of Mn^4+^ with a separation of 11.9 eV (Fig. 2g)^50^. Raman spectrum of MnO_2_ with the characteristic peaks at 491, 568 and 635 cm^−1^ indicates the birnessite-type crystalline structure (Fig. 2h)^51^. The binder-free procedure facilely realizes the integration of nanoporous metal/oxide composites with current collectors without any additional contact resistance while the bicontinuous nanoporous channels and Cu skeleton facilitate ion and electron transport kinetics. These advantages enlist the hybrid electrodes to not only exhibit exceptional mechanical flexibility and stability (Fig. 1e, Supplementary Fig. S1e), but minimize the primary resistances in the entire hybrid electrodes for the enhanced charge storage.

**Figure 3:**
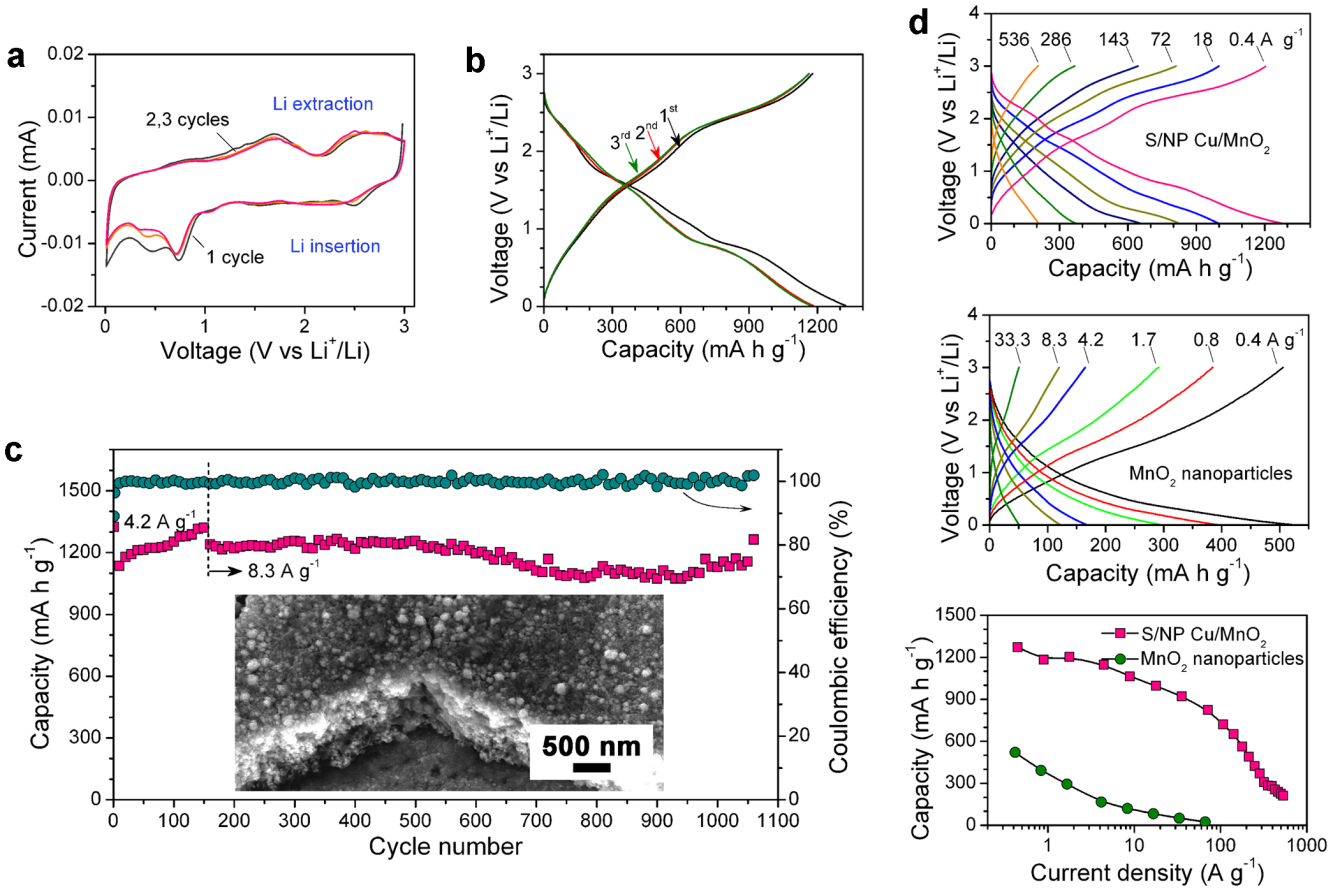
Electrochemical performance. (a), CV curves at a scan rate of 0.2 mV s^−1^ and, (b), galvanostatic charge/discharge profiles at the current rate of 0.8 A g^−1^ for the initial three cycles of the S/NP Cu/MnO_2_ electrode in the potential window of 0.01–3 V. (c), The reversible capacity retention and Coulombic efficiency of the S/NP Cu/MnO_2_ hybrid bulk electrode during the first cycles at 4.2 A g^−1^ followed by a higher current density of 8.3 A g^−1^. Inset: SEM micrograph of a S/NP Cu/MnO_2_ hybrid bulk electrode after charge/discharge cycling. (d), Charge/discharge profiles of the S/NP Cu/MnO_2_ electrode (top) and MnO_2_ nanoparticles on Cu foil (middle) and the specific capacities of their constituent MnO_2_ as a function of current density (bottom).

**Figure 4:**
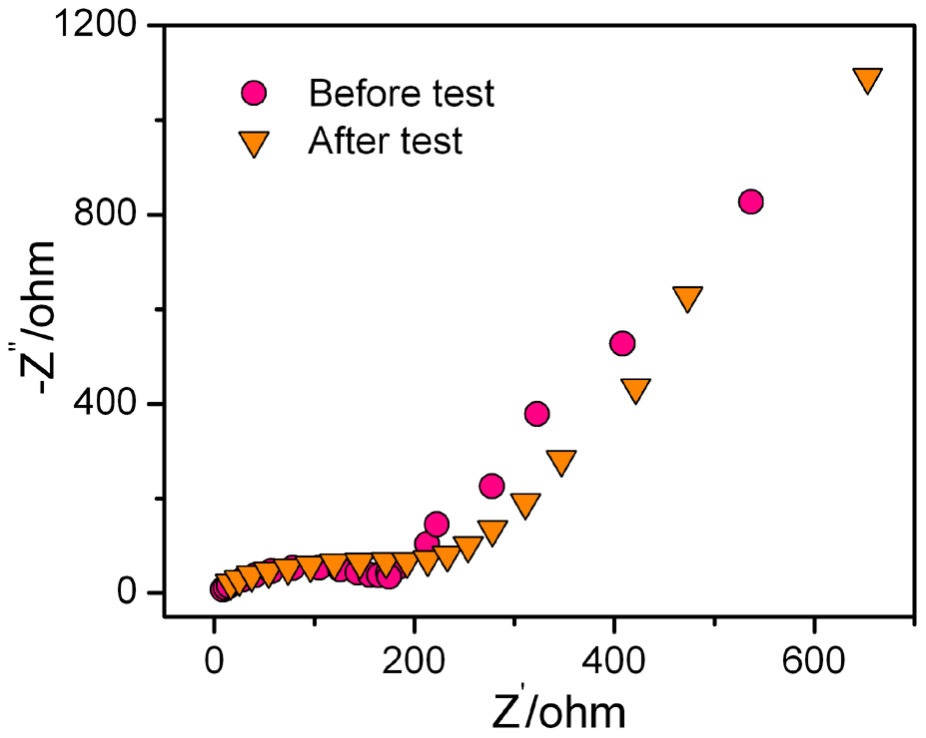
Electrochemical impedance spectroscopy measurements. EIS spectra for the cell with S/NP Cu/MnO_2_ electrode before and after the rate performance test.

The electrochemical properties of the S/NP Cu/MnO_2_ hybrid bulk electrode are tested in a two-electrode configuration (Figs. 1f, g), in which a lithium foil and a porous polymer film are used as a counter electrode and a separator, respectively. Figure 3a shows cyclic voltammetry (CV) curves of the first three cycles of the S/NP Cu/MnO_2_ hybrid electrode in the range of 0.01 and 3 V (*vs.* Li^+^/Li) at a scan rate of 0.2 mV s^−1^. Accompanied by a pseudocapacitive process 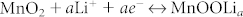
11,17,50, two pairs of cathodic/anodic peaks at 0.73/2.5 and 0.46/1.7 V in the first cycle correspond to the reduction/oxidation reactions of 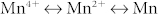 during the Li insertion/extraction, i.e., 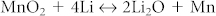
7,8. The current of two cathodic peaks in the sequent cycles slightly reduces most likely due to formation of solid-electrolyte-interphase (SEI) layer on the electrode surface during the first discharge step^8^,^19^,^40^. As a consequence, the first discharge and charge steps deliver specific capacities of ~1324 and ~1179 mA h g^−1^, respectively, with a Coulombic efficiency of ~89% (Fig. 3b). The most overlapping of the sequent CV and charge/discharge curves implies an outstanding reversibility of conversion reaction of MnO_2_ during Li insertion/extraction (Figs. 3a, b) although it is probably accompanied by the oxidation of the intimate contact Cu layer^52^. The negligible influence of Cu oxidation on the charge/discharge behavior of the whole electrode is demonstrated by electrochemical impedance spectroscopy (EIS) measurements, which are performed on the cell with the S/NP Cu/MnO_2_ electrode before and after cycling test (Fig. 4). As shown in the Nyquist plot, both EIS spectra exhibit a characteristic semicircle in the high- and middle-frequency range, followed by a inclined line in the low-frequency range, with inconspicuous shape variations. This suggests the exceptional retention of charge transfer and Li^+^ ion diffusion in the S/NP Cu/MnO_2_ electrode during cycling as a result of the stable architecture (**inset of **Fig. 3c), of which 3D bicontinuous nanoporous channels with extremely large specific surface area of electrode/electrolyte interface offers the short ion diffusion and the Cu/MnO_2_ network seamlessly integrated with Cu current collector facilitates electron transport. At the low frequency, the inclined lines of both fresh and tested S/NP Cu/MnO_2_ electrodes exhibit the similar slopes, revealing the almost same solid-state diffusion of Li^+^ ions in electrodes^53^,^54^. While the small difference of semicircles at the middle frequency is due to the formation of SEI layer after the discharge, giving rise to a slightly larger charge transfer resistance at the electrode/electrolyte interface^53^,^54^. These primary properties enable excellent cyclability of the S/NP Cu/MnO_2_ electrode, which is further verified by the cycling performance in the voltage range of 0.01–3 V vs. Li^+^/Li (Fig. 3c, Supplementary Fig. S9). After the first cycle the Coulombic efficiency increases to more than 98%, and the discharge capacity gradually increases from ~1135 mA h g^−1^ to ~1320 mA h g^−1^ due to the improvement of lithium ion accessibility in the electrode during the initial 150 charge/discharge cycles at the current density of 4.2 A g^−1^ 55. Even the current density is increased to 8.4 A g^−1^, a stably reversible capacity more than 1100 mA h g^−1^ can be maintained for ~1000 cycles with the Coulombic efficiency of ~99% (Fig. 3c, Supplementary Fig. S9). The attractive capacity retention results from the solid Cu/MnO_2_ interface (**inset of **Fig. 3c) and the stable 3D nanoporous architecture, which affords enough space to accommodate the volume changes during the charge/discharge processes^1^,^2^,^3^,^4^,^8^,^22^,^23^.

Whereas MnO_2_ is known to suffer from poor conductivity^1^,^8^,^9^,^17^,^40^, its rate capability is substantially enhanced by the seamlessly integrated architecture of S/NP Cu/MnO_2_ (Fig. 3d). The discharge capacity of the constituent MnO_2_ in the hybrid bulk electrodes reaches ~1270 mA h g^−1^ at low rate such as 0.4 A g^−1^, and retains ~78% (~996 mA h g^−1^) and ~51% (~652 mA h g^−1^) at the exceptionally high discharge rates of 18 A g^−1^ and 143 A g^−1^ (corresponding to full discharge in 14 and 116 C, Supplementary Fig. S10), respectively. Even at 377 C the hybrid electrode delivers the capacity of ~240 mA h g^−1^ (Supplementary Fig. S10), and the discharge capacity reverts to ~1270 mA h g^−1^ when the current density returns to 0.4 A g^−1^ (Supplementary Fig. S11). Moreover, the further increase of the MnO_2_ loading by thickening nanoporous Cu/MnO_2_ layer to 1.2 μm does not lead to remarkable capacity fading of the constituent MnO_2_ at high current densities (Supplementary Fig. S12). The rate performance is in distinct contrast with that of MnO_2_ nanoparticles (similar to those incorporated into S/NP Cu skeleton) supported by Cu foil (the middle plot of Fig. 3d, Supplementary Fig. S13) and directly grown onto Cu foil at the same electroless plating conditions (Supplementary Fig. S14). For comparison, the rate capabilities of carbon nanotubes/MnO_2_^8^, carbon nanohorns/MnO_2_^39^, graphene/MnO_2_^40^,^41^, graphene/Mn_3_O_4_^9^ and carbon nanotube/CuO^10^ nanocomposites are also included in Supplementary Fig. S14. Although low-dimensional nanostructures can shorten solid-state ion diffusion length^2^,^3^,^7^,^20^ and conductive agents can ameliorate the electron transport^4^,^8^,^9^,^10^,^13^,^40^, the entire electrodes assembled with these nanostructures using a conventional approach exhibit much lower capacity and remarkable fading for metal oxide-based composite nanostructures even at the current densities below 10 A g^−1^ (Fig. 3d, Supplementary Figs. S11 and S14)^8^,^9^,^40^. This further highlights the merits of S/NP Cu architecture in the high-performance lithium storage of S/NP Cu/MnO_2_ hybrid electrodes. To assess the contribution of total capacity from oxides that are produced from the oxidation of NP Cu skeleton^29^,^30^,^31^,^32^,^33^,^34^,^35^,^36^ and the remaining Mn in KMnO_4_ solution^26^, the charge/discharge profiles of bare and KMnO_4_-treated NP Cu foils are measured at the same conditions (Supplementary Figs. S15 and S16). It demonstrates that at the low current density of 0.02 mA cm^−2^, the areal capacity of the produced oxides is ~0.0074 mA h cm^−2^, accounting for only ~6.7% of the total capacity of S/NP Cu/MnO_2_. While the current density increases to 2 mA cm^−2^, this contribution further decreases to less than 1% (Supplementary Fig. S17).

## Discussion

**Figure 5:**
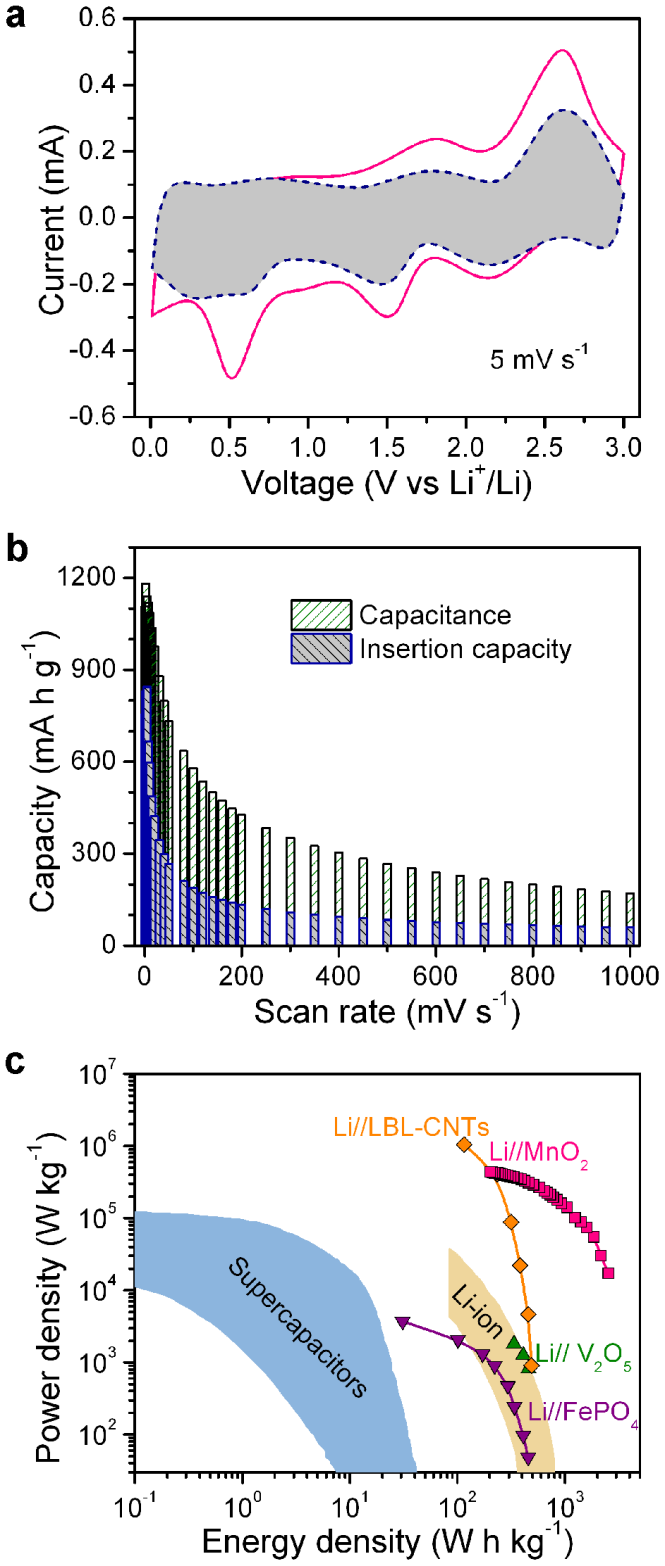
Capacitance and Li-insertion/extraction contributions to charge storage/delivery and the Ragone plot. (a), Cyclic voltammetric responses for S/NP Cu/MnO_2_ hybrid bulk electrode at a scan rate of 5 mV s^−1^. The Li-insertion/extraction contribution is evaluated according to Eq. (1) and shown by the shaded region. (b), Capacitance and Li-insertion capacity for S/NP Cu/MnO_2_ electrode as a function of scan rate. (c), The Ragone plot of the constituent MnO_2_ nanoparticles in S/NP Cu/MnO_2_ electrode, comparing with the energy and power densities of the active materials in the composites of CNT/FePO_4_^3^, functionalized LBL-CNTs^14^, graphene/V_2_O_5_^58^ (only the active materials are included in the weight). The values of Li-ion nanoporous carbon/LiFePO_4_^14^,^59^, LiNi_0.5_Mn_0.5_O_2_^14^,^60^, and supercapacitive electrodes^11^,^14^ are also included for comparison.

To analyze the charge storage of S/NP Cu/MnO_2_ hybrid electrodes, the voltammetric behavior at various scan rates (*v*) is reexamined in the voltage range of 0.01–3 V (vs. Li^+^/Li) (Supplementary Fig. S18a), wherein the current response (*i*) at a fixed voltage (*V*) is described as the combination of capacitive effect (*k*_1_*v*) and diffusion-controlled Li insertion/extraction (*k*_2_*v*^1/2^)^56^,^57^, i.e., 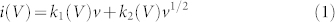
Their different scan-rate dependence of current response is employed to distinguish the fraction of current arising from capacitive and Li insertion processes by determining both *k*_1_ and *k*_2_ in the light of the methodology proposed in Refs. 56 and 57. For analytic purpose, Eq. (1) can be rearranged to *i*(*V*)/*v*^1/2^ = *k*_1_(*V*)*v*^1/2^ + *k*_2_(*V*), according to which the values of *k*_1_(*V*) and *k*_2_(*V*) are obtained from the slope and the y-axis intercept point for *i*(*V*)/*v*^1/2^ as a linear function of *v*^1/2^, respectively, at each fixed potential^56^,^57^. Figure 5a shows the typical voltage profile for the insertion/extraction current (shaded region) approximately estimated according to Eq. (1), in comparison with the total current of S/NP Cu/MnO_2_ electrode at a scan rate of 5 mV s^−1^, wherein the current of Li insertion/extraction is estimated in terms of the equation *i*_insertion_(*V*) = *k*_2_(*V*)*v*^1/2^. It illustrates that the total stored charge consists of both Li insertion and capacitive processes and their tradeoff depends on the scan rate (Fig. 5b)^56^,^57^. For relatively low scan rates (<10 mV s^−1^), the insertion/extraction process delivers >60% of the total capacities; whereas at higher scan rates, the insertion capacity drops to ~32% and the capacitive charge storage primarily resulting from pseudocapacitive contribution becomes dominant (~68%) (Fig. 5b)^17^ although the double-layer capacitance of bare S/NP Cu decreases from ~13% to ~6% (Supplementary Fig. S17). The unique feature combining both battery- and supercapacitor-like behaviors in this hybrid bulk electrode offers ultrahigh rate capability without remarkable fading of capacity in the 0.01–3 V range (Fig. 3d, Supplementary Figs. S10 and S11)^4^,^14^, and enlists the constituent MnO_2_ to exhibit high gravimetric energy (200 W h kg^−1^) delivered at an exceptionally high power of 430 kW kg^−1^ in Li//S/NP Cu/MnO_2_ cells, much higher than the active materials in CNT/FePO_4_^3^, functionalized LBL-CNTs^14^, graphene/V_2_O_5_^58^, nanoporous carbon/LiFePO_4_^14^,^59^, and LiNi_0.5_Mn_0.5_O_2_^14^,^60^, as well as supercapacitive electrodes^11^,^14^ (Fig. 5c). Even the mass of nanoporous Cu layer is included, the power and energy densities of NP Cu/MnO_2_ reach maxima of ~74 kW kg^−1^ and ~360 W h kg^−1^ (Supplementary Fig. S19). Although MnO_2_ has intrinsically low conductivity that limits its charge/discharge rate, the charge storage performance can be significantly enhanced by the dual mechanism of capacitive storage and Li insertion in the unique integration of S/NP Cu/MnO_2_ architecture, wherein (i) the intimate Cu/MnO_2_ interface accelerates the electron transport between the nanocrystalline MnO_2_ and Cu ligaments and stabilizes the hybrid structure; (ii) the interconnected-pore and -ligament Cu network shortens the ion diffusion path length and improves the electrical conductivity, respectively; (iii) the seamless integration minimizes the contact resistances between NP Cu/MnO_2_ and copper current collector; (iv) the good nanoporous structure provides a large specific surface area to facilitate the full use of the capacitive charge storage of MnO_2_ while also accommodating large volume changes on Li insertion/extraction of MnO_2_ for the improved cyclability. These four advantages make the high-rate feature comparable to that of LBL-CNT electrodes assembled with the functionalized CNTs by layer-by-layer technique, which stores charge by only surface-redox process^14^.

In summary, we have developed hybrid bulk electrodes with seamlessly integrated nanoarchitecture of S/NP Cu/MnO_2_ by a procedure combining physical deposition with chemical dealloying and modified electroless plating. The hybrid bulk electrodes store/deliver high energy at ultrahigh rates with excellent stability by a dual mechanism of pseudocapacitive and Li insertion/extraction processes, making the NP copper/oxide hybrids promising use in miniaturized devices for high power- and energy-density applications. Such exceptional charge-storage performance results from the intimate contact of Cu/MnO_2_ and the unique S/NP integration architecture, simultaneously minimizing the primary resistances during charge and discharge and accommodating large volume change during Li insertion/extraction.

## Methods

### Fabrication of seamless S/NP Cu/MnO_2_ hybrid bulk electrodes

Seamlessly integrated S/NP Cu foils were fabricated by a combination of physical deposition and chemical dealloying. Cu_30_Mn_70_ (atomic ratio) alloy films with a thickness of ~800 nm were deposited on solid Cu foils with dimensions of ~3 cm × 2 cm × 10 μm by direct current magnetron sputtering with a power of 200 W for 20 min at room temperature. The solid Cu foils were cleaned thoroughly with acetone, 1 M HCl solution and deionized water (18.2 MΩ·cm) before the physical deposition. Nanoporous Cu layer on the solid Cu substrate was produced by chemically dealloying Cu_30_Mn_70_ alloy film for 5 h at room temperature in 10 mM HCl solution that was firstly bubbled by N_2_ gas for 30 min^44^,^45^,^46^. The residual acid in nanoporous copper was removed by N_2_ bubbled water rinsing. MnO_2_ nanocrystals were plated onto the clean S/NP copper foils by a modified electroless plating technique in the aqueous mixture of 5 mM KMnO_4_ and 10 mM KOH for 30 minutes at room temperature under the gas reagent of hydrazine (N_2_H_4_). The complete immersion of S/NP Cu foils in the aqueous solution allows the uniform growth of MnO_2_ nanocrystals along the Cu ligaments. All specimens are dried in vacuum (~10^−4^ torr) after thorough water rinsing. The mass of loading MnO_2_ is calculated according to the mass of nanoporous Cu skeleton and the weight ratio of nanoporous Cu/MnO_2_ determined by EDS measurements (Fig. S2c).

### Structural characterization

The microstructure and chemical composition of the specimens were investigated using a field-emission scanning electron microscope (JEOL 6700F) equipped with an X-ray energy-dispersive microscopy (EDS), and a transmission electron microscope (JEOL JEM-2100F, 200 keV). X-ray photoelectron spectroscopy (XPS, AxIS-ULTRA-DLD) with Al Kα (mono) anode at energy of 150 W in a vacuum of 10^−7^ Pa. X-ray diffraction measurement was carried out on a D/Max2500pc diffractometer using Cu *Kα* radiation. Raman spectrum was collected using a micro-Raman spectrometer (Renishaw) with a laser of 532 nm wavelength.

### Construction of the lithium ion battery and electrochemical measurement

Coin-type cells (2016) were assembled in an argon-filled dry glove box (both moisture and oxygen levels were kept below 1 ppm) using the S/NP Cu/MnO_2_ as the positive electrode and the Li foil as the negative electrode. Both positive and negative electrodes were electronically separated by Celgard 2400 film in non-aqueous electrolyte (1 M LiPF_6_ in 1:1:1 volume ratio mixture of ethylene carbonate (EC), ethylmethyl carbonate (EMC) and dimethyl carbonate (DMC)). CV was performed on an IVIUM electrochemical analyzer, and the charge/discharge measurements were carried out on a battery test system in the voltage range between 0.01 and 3.0 V (vs. Li^+^/Li) at room temperature. EIS measurements were carried out over the frequency range from 10 mHz to 10 kHz with an amplitude of 5 mV.

## Author Contributions

C.H., X.Y.L. and Q.J. conceived and designed the experiments. C.H., X.Y.L., G.F.H. and Y.Q.L. carried out the fabrication of materials and performed electrochemical measurements. L.Z., Z.W., Y.F.Z. contributed to microstructural characterizations. M.Z. J.C.L. and J.S.L. provided helps in the experiments. C.H., X.Y.L. and Q.J. wrote the paper, and all authors discussed the results and commented on the manuscript.

## Supplementary Material

Supplementary Information Supplementary information for publication
