# Supplementary material for: Integrated Solid/Nanoporous Copper/Oxide Hybrid Bulk Electrodes for High-performance Lithium-Ion Batteries
Source: Sci Rep. 2013 Oct 7;3:2878. doi: 10.1038/srep02878 (PMC3791456; doi:10.1038/srep02878)
Supplement: Supplementary Information — for publication [file srep02878-s1.pdf]

Supplementary Information

for

Integrated Solid/Nanoporous Copper/Oxide Hybrid Bulk Electrodes for High-performance Lithium-Ion Batteries

Chao Hou,[†] Xing-You Lang,[†]* Gao-Feng Han, Ying-Qi Li, Lei Zhao, Zi Wen, Yong-Fu Zhu, Ming Zhao, Jian-Chen Li, Jian-She Lian, Qing Jiang*

Key Laboratory of Automobile Materials (Jilin University), Ministry of Education, and School of Materials Science and Engineering, Jilin University, Changchun 130022, China

[†] These authors contributed equally to this work.

* Correspondence and requests for materials should be addressed to X.Y.L. (email: xylang@jlu.edu.cn) or Q.J. (email: jiangq@jlu.edu.cn).

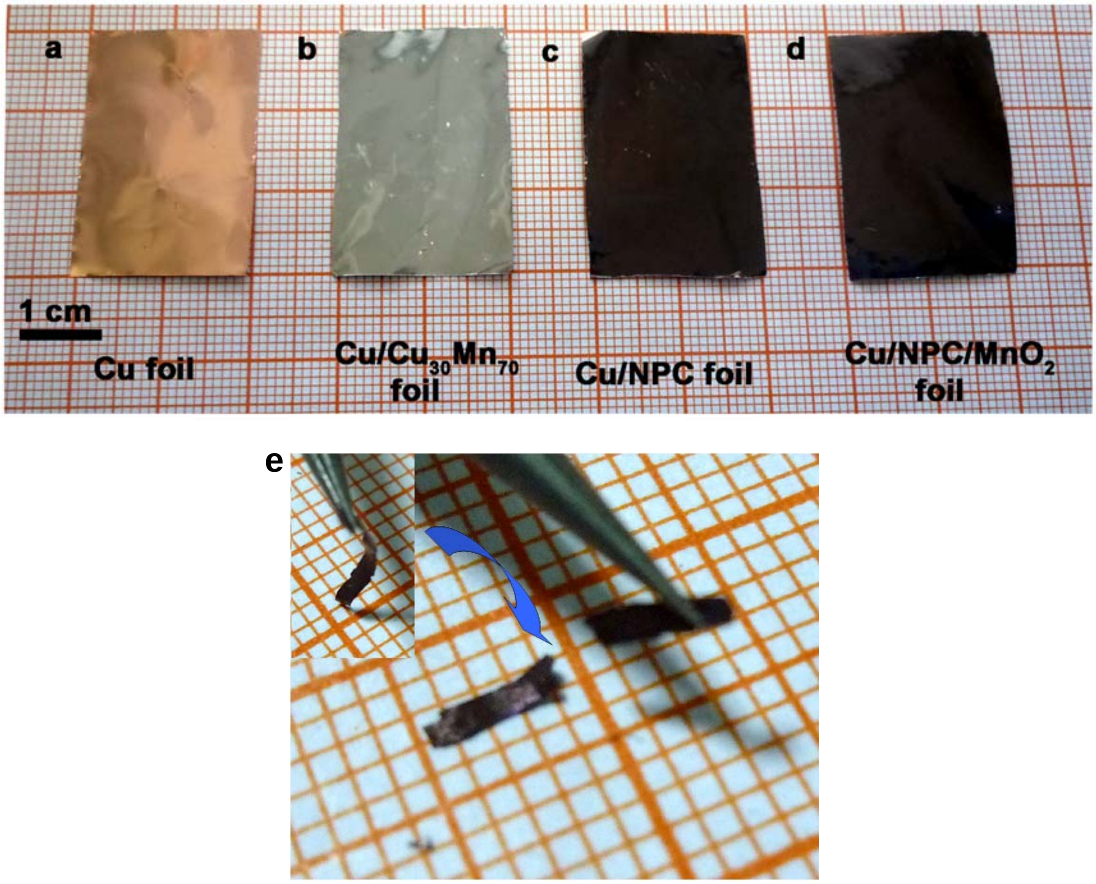

Supplementary Figure S1. Photographs of (a) copper foil, (b) copper foil supported $Cu_{30}Mn_{70}$, (c) solid/nanoporous copper foil, (d) solid/nanoporous copper/MnO_2 hybrid foil, showing excellent mechanical flexibility and stability in comparison with and (e) the fragile NP Cu ribbon.

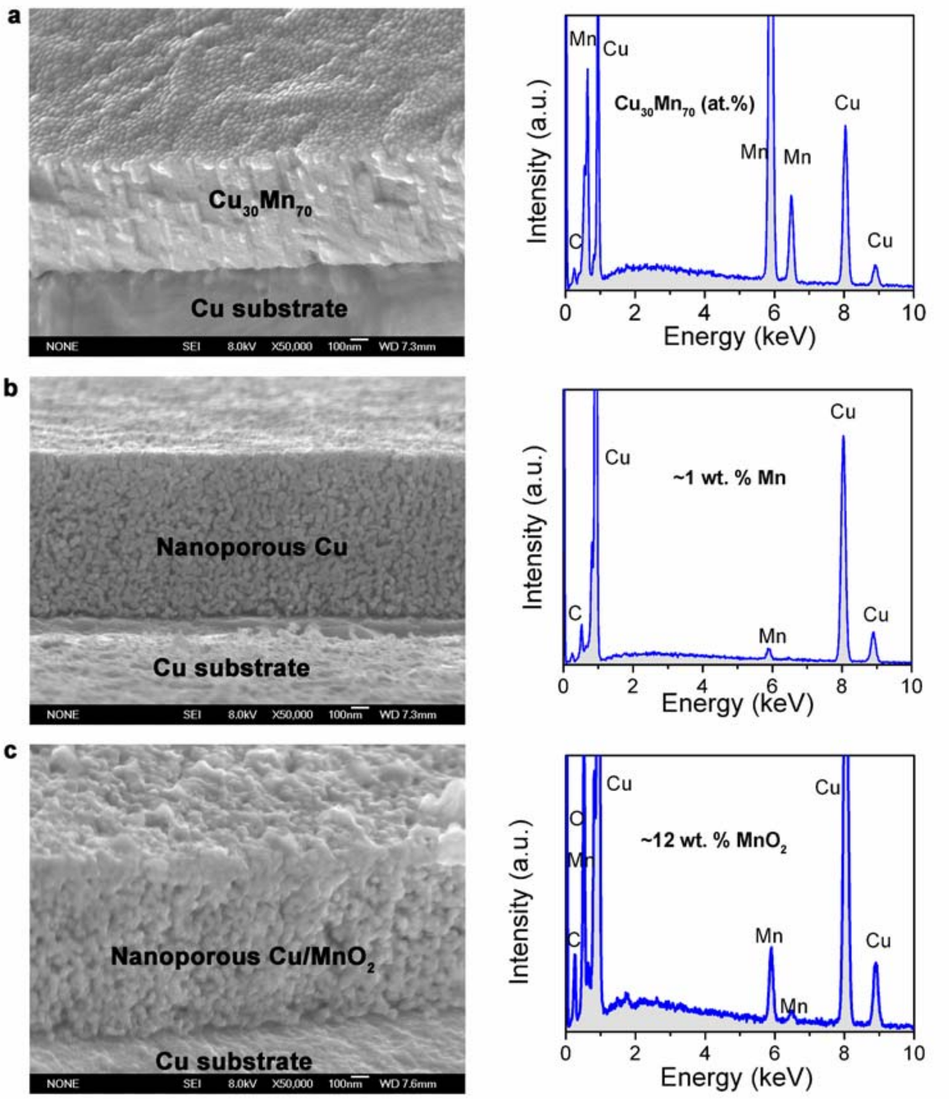

Supplementary Figure S2. Typical SEM images and EDS spectra of (a) copper foil supported $Cu_{30}Mn_{70}$ film, (b) solid/nanoporous Cu foil after chemical dealloying, (c) solid/nanoporous Cu/MnO_2 foil.

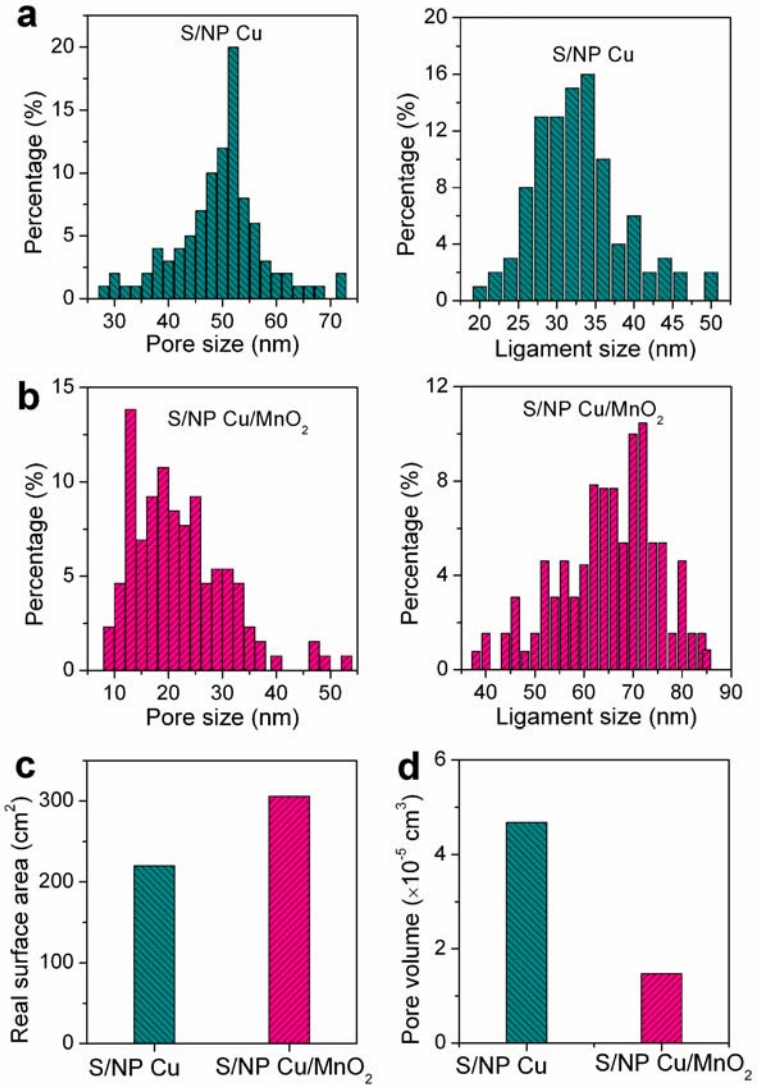

Supplementary Figure S3. Distributions of Cu ligament and nanoporous channel sizes of (a) S/NP Cu and (b) S/NP Cu/MnO_2 foils. (c) Real surface area and (d) pore volumes of S/NP Cu and S/NP Cu/MnO_2 foils.

On the basis of the assumption that each surface Cu atom corresponds to one chemisorbed O atom, the real surface area of S/NP Cu foil ($A_{\text{real,S/NP Cu}}$) is determined by measuring the charges ($Q_{\text{Cu,O}}$) from the Cu (0) to Cu (Ⅰ) oxidation peak of its CV curve, i.e., $A_{\text{real,S/NP Cu}} = Q_{\text{Cu,O}}/Q_{\text{Cu,s}}$, where $Q_{\text{Cu,s}}$ = 360 μC cm^{-2} is the charges of a smooth Cu surface[1,2]. According to the CV curve of S/NP Cu foil in 0.1 M KOH aqueous solution at a scan rate of 1 mV s^{-1}, $A_{\text{real,S/NP Cu}}$ = 222 cm^{-2} with $Q_{\text{Cu,O}}$ = 0.08 C. While the surface area of S/NP Cu/MnO_2 electrode is determined by capacitance measurement method according to the equation $A=i/(vC_F)$[2,3]. Here, i is the capacitive

current at voltage of -0.4 V *vs* Ag/AgCl, *v* is the scan rate of CV curves that are recorded in the 0.1 M KOH electrolyte (5-100 mV s^{-1}), C_F is the surface capacitance of MnO_2 with $C_F \approx 60$ μF cm^{-2} for the ideally smooth oxide surface[2]. Thus the real surface area of the S/NP Cu/MnO_2 electrode is 306 cm^2.

To calculate the pore volumes, the SEM micrographs of S/NP Cu and S/NP Cu/MnO_2 are analyzed with ImageJ digital imaging software (NIH shareware, http: rsb.info.nih.gov/ij/index.html)[4,5]. Firstly, the gray-scale image is segmented into binary image composed of only black pores and white ligaments. Then the percent porosities (ratio of dark area to total image area) is calculated with ″particle analysis″ algorithm built-in ImageJ. The percent porosities (C_P) are firstly evaluated to be 58.505% and 18.393% for S/NP Cu and S/NP Cu/MnO_2 specimens, respectively. The pore volumes (V_p) of S/NP Cu and S/NP Cu/MnO_2 are calculated in terms of the equation $V_p = C_P V$, where V is the real volume of nanoporous layer.

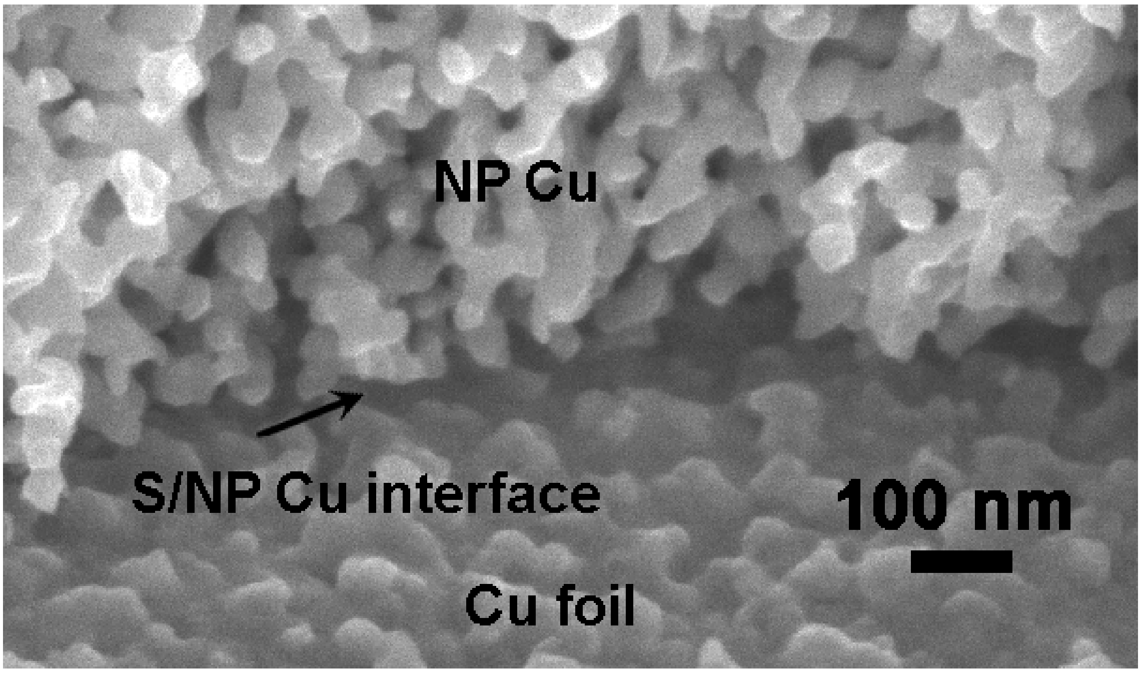

Supplementary Figure S4. High-resolution SEM image of seamlessly integrated solid/nanoporous Cu interface.

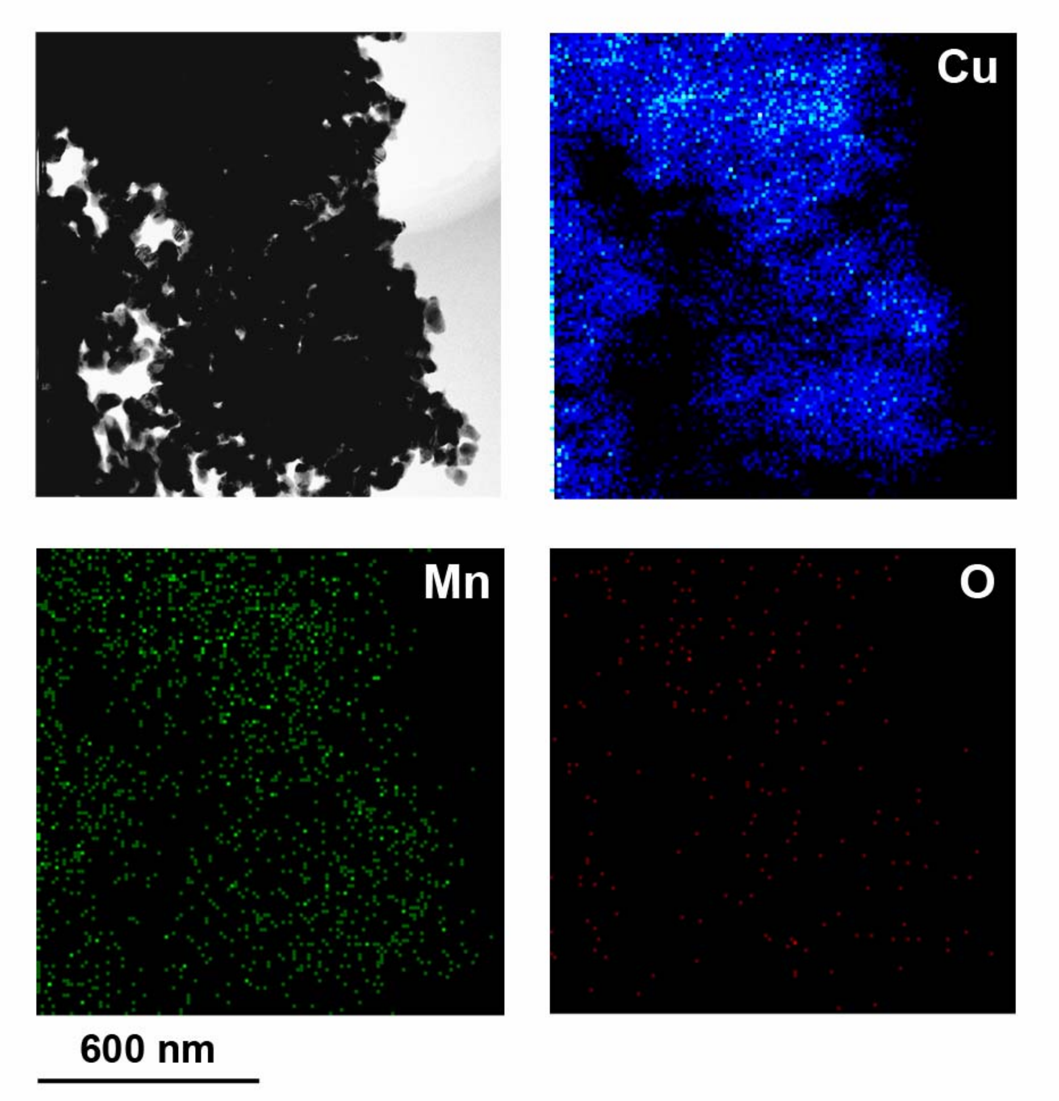

Elements	Composition (wt. %)
Cu	98.61
Mn	1.14
O	0.25

Supplementary Figure S5. Scanning TEM energy disperse spectroscopy (STEM-EDS) chemical mapping of as-dealloyed NP Cu, showing the negligible Mn atoms and Cu oxides in Cu skeletons.

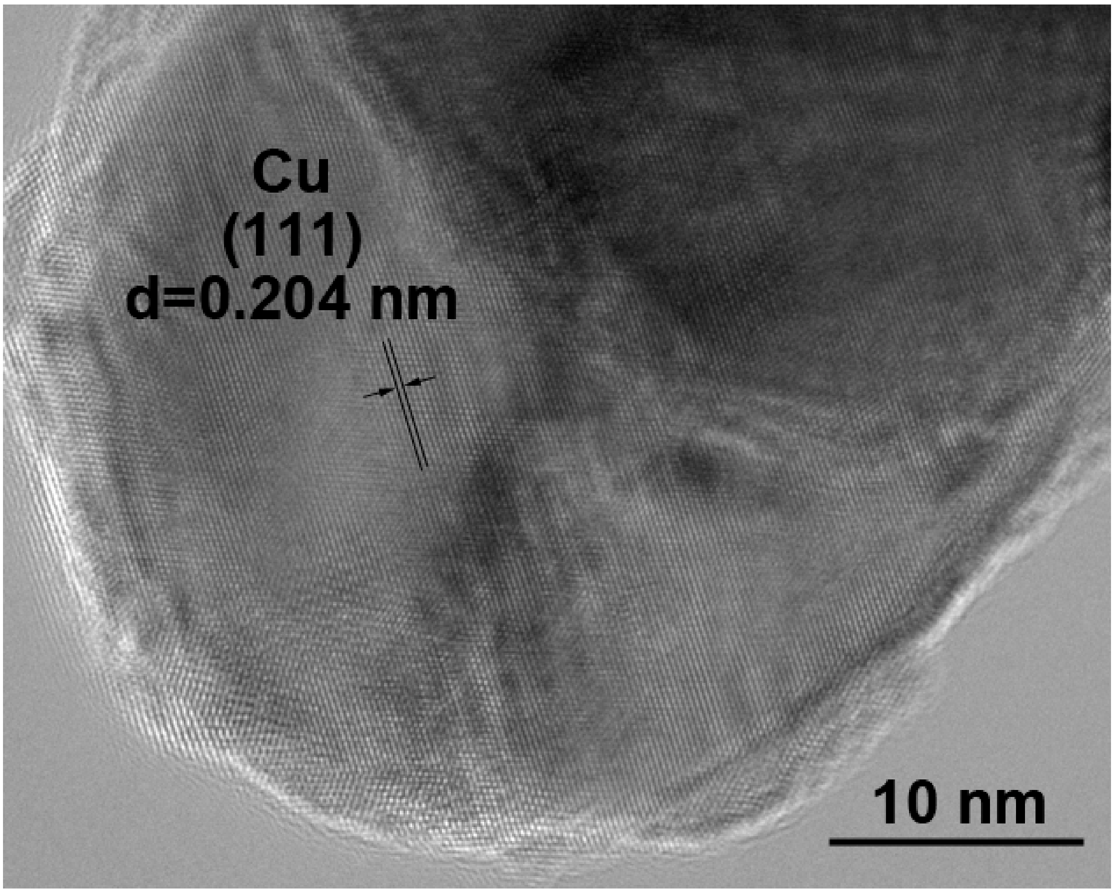

Supplementary Figure S6. HRTEM image of NP Cu with the plane of Cu (111).

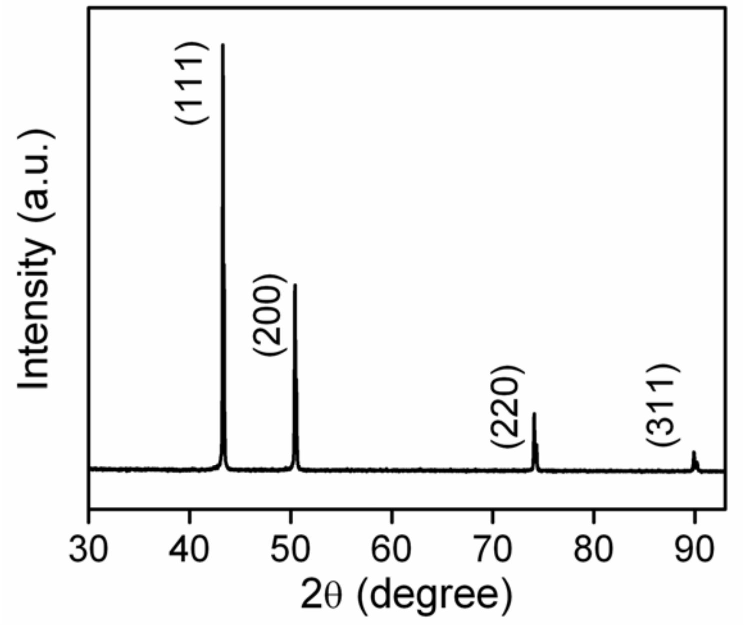

Supplementary Figure S7. XRD pattern of the S/NP Cu foil. The obvious diffraction peaks corresponds to the (111), (200), (220) and (311) planes of Cu (JCPDS 04-0836).

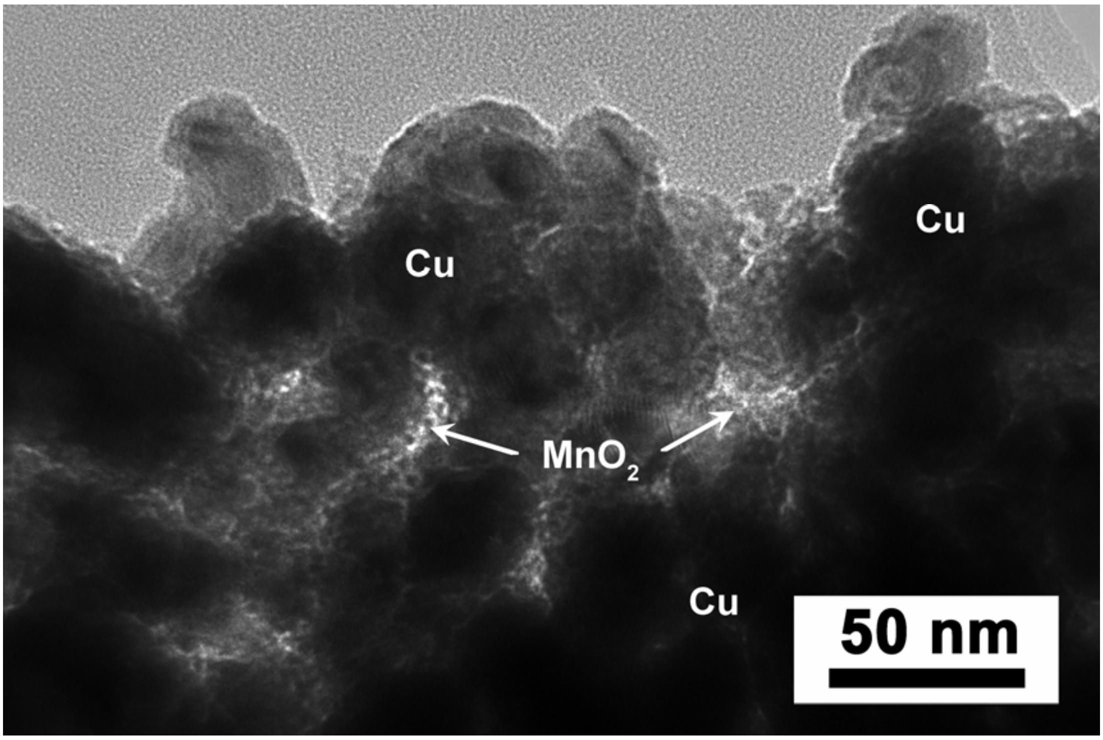

Supplementary Figure S8. Low-magnification TEM micrograph of S/NP Cu/MnO_2 hybrid.

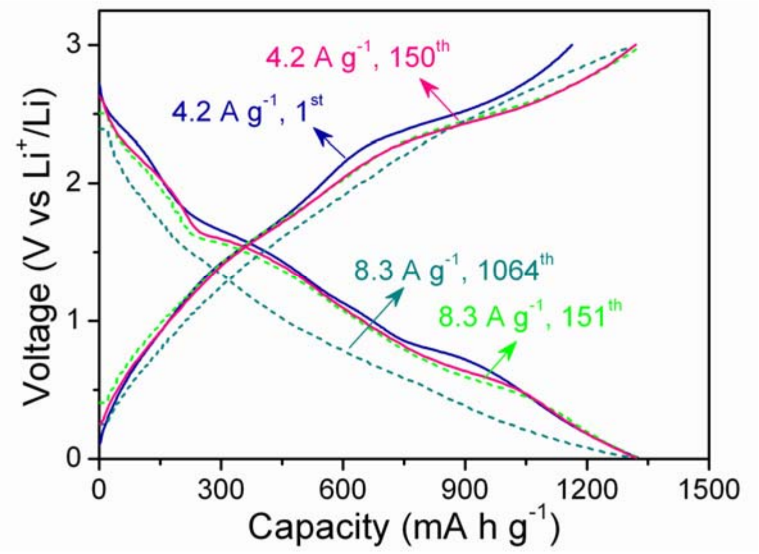

Supplementary Figure S9. 1st, 150th and 151th, 1064th charge/discharge curves of the S/NPG Cu/MnO_2 bulk electrode at the current densities of 4.2 and 8.3 A g^{-1}, respectively.

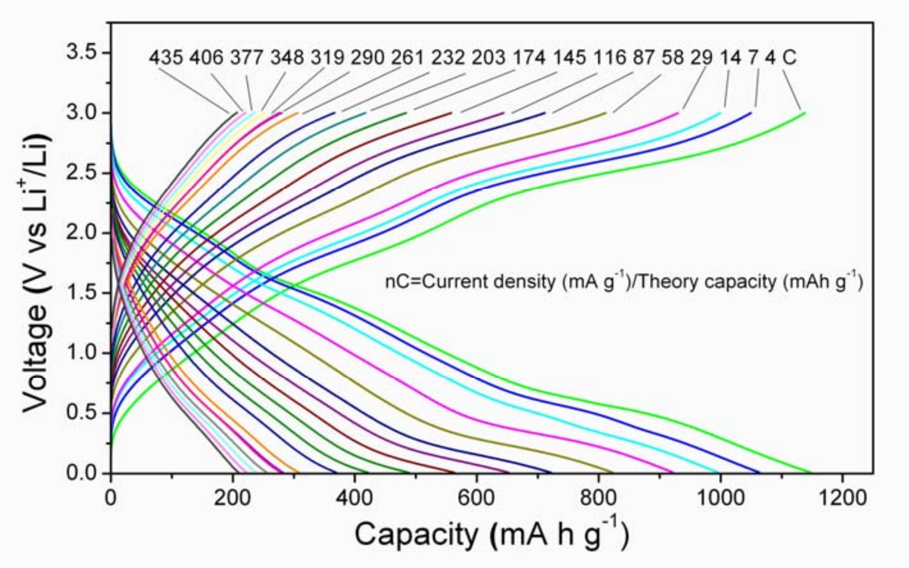

Supplementary Figure S10. Charge/discharge profiles of the S/NP Cu/MnO_2 bulk electrode at a wide range of *C* rates (4C-435C) between 0.01 and 3.0 V.

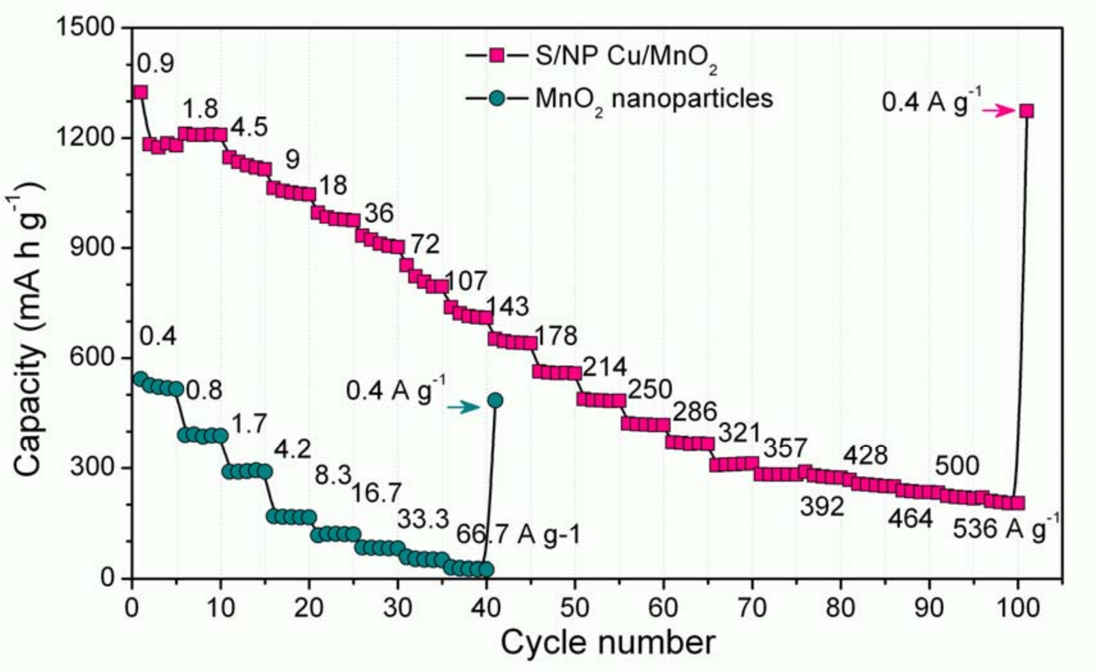

Supplementary Figure S11. Comparison of rate capability between the electrodes based on S/NP Cu/MnO_2 hybrid bulk and MnO_2 nanoparticles at various current densities.

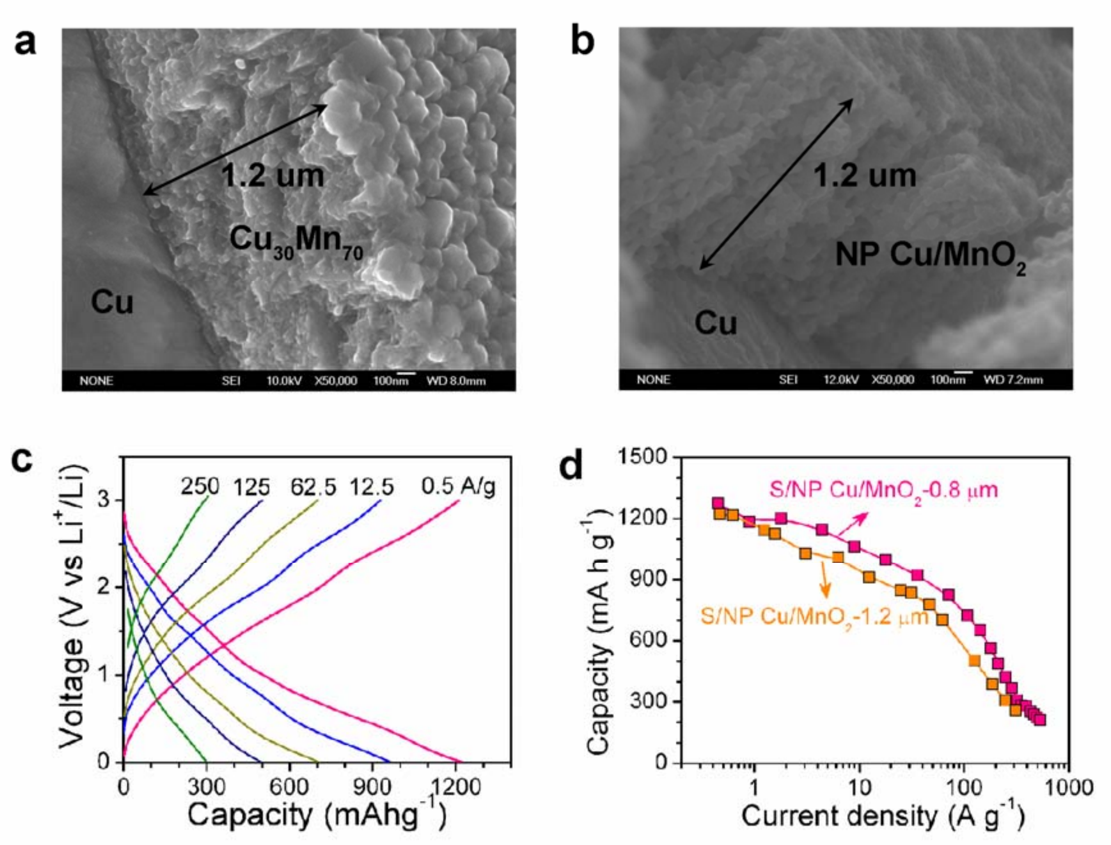

Supplementary Figure S12. Typical cross-sectional SEM images for (a) $Cu/Cu_{30}Mn_{70}$ and (b) S/NP Cu/MnO_2 hybrid with the thickness of ~1.2 μm. (c) Representative charge/discharge curves 1.2-μm-thick S/NP Cu/MnO_2 hybrid electrode at various current densities. (d) Capacity vs current density plot for S/NP Cu/MnO_2 hybrid electrodes with the thicknesses of ~0.8 and ~1.2 μm. Here the 1.2-μm-thick $Cu_{30}Mn_{70}$ film is magnetron sputtered onto Cu foil with a power of 200 W for 40 minutes.

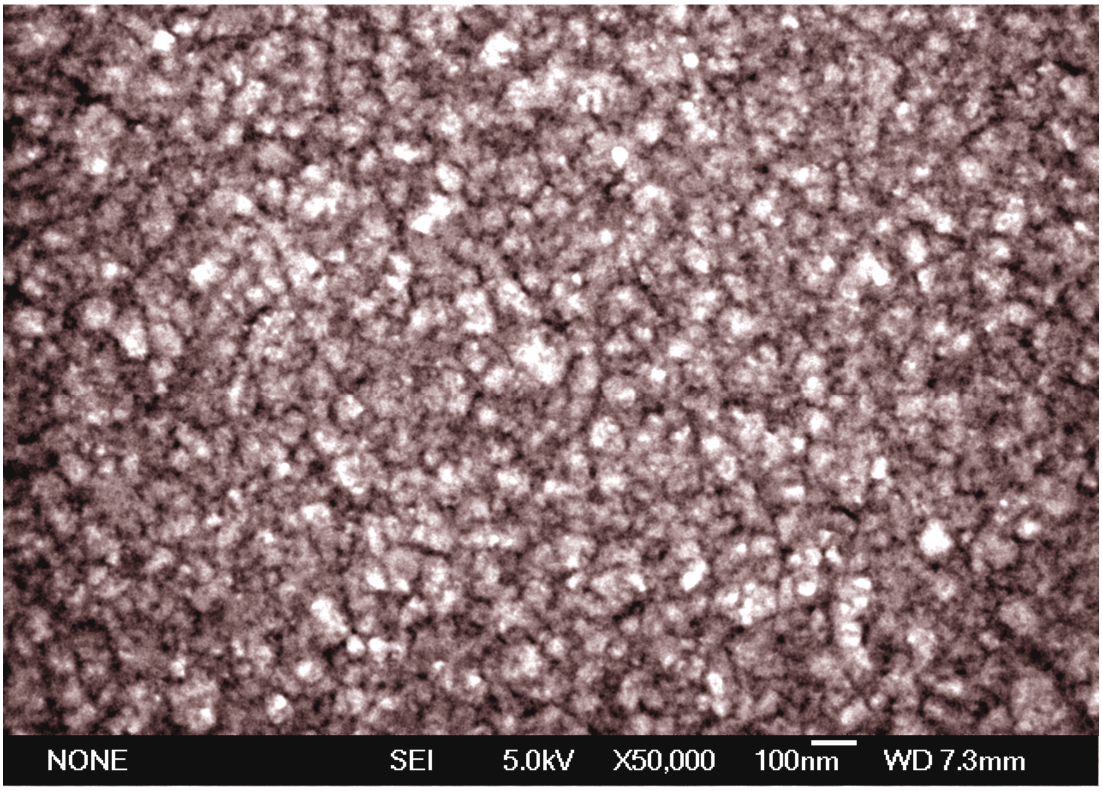

Supplementary Figure S13. Representative SEM image of MnO_2 nanoparticles supported by Cu foil.

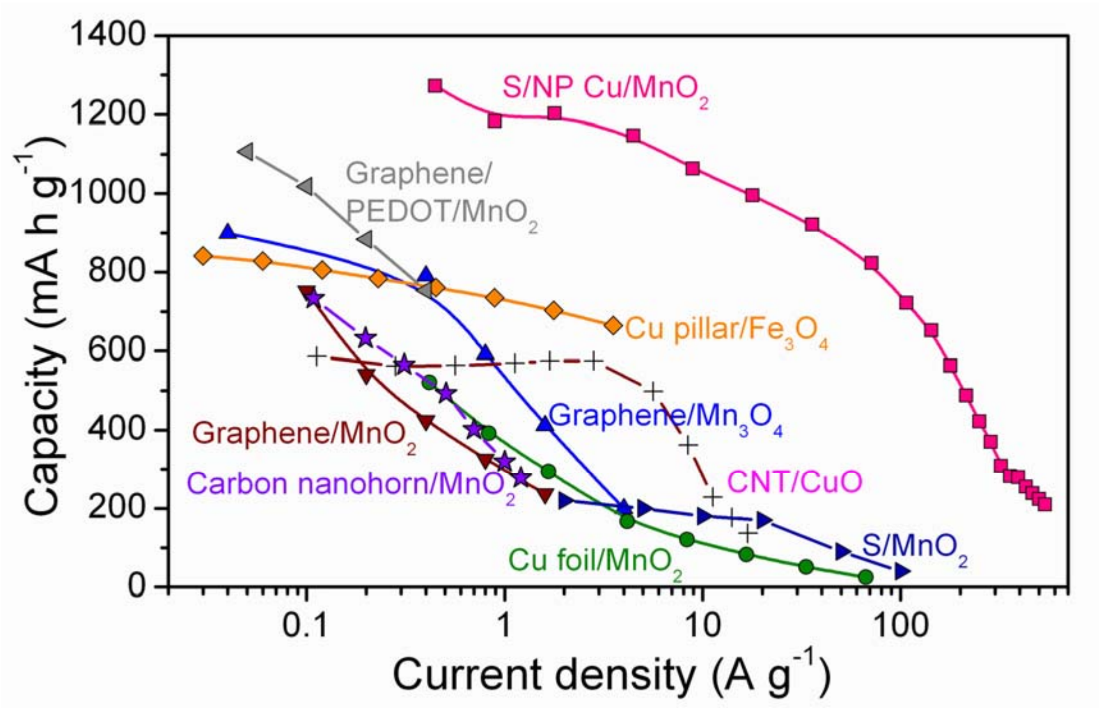

Supplementary Figure S14. Comparison of rate capabilities between S/NP Cu/MnO_2 hybrid bulk electrode (□) and other metal oxide-based electrodes assembled with low-dimensional nanostructures such as graphene/MnO_2 (▽)[37], graphene/PEDOT/MnO_2 (◁)[38], carbon nanohorn/MnO_2 (☆)[36], graphene/Mn_3O_4 (△)[9], carbon nanotube/CuO (+) [10], Cu pillar/Fe_3O_4 (◇) [23], and MnO_2 nanoparticles supported by Cu foil (○) and directly grown on Cu foil (▷).

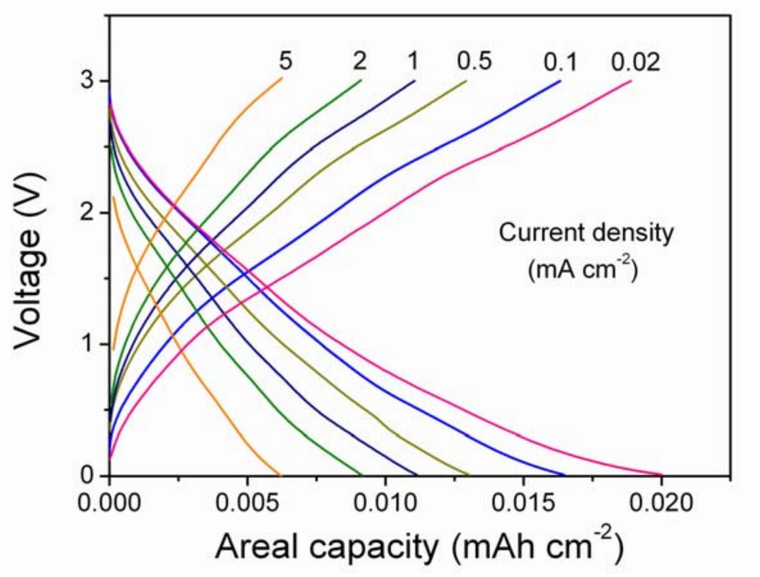

Supplementary Figure S15. Charge/discharge profiles of S/NP Cu electrode at various current densities.

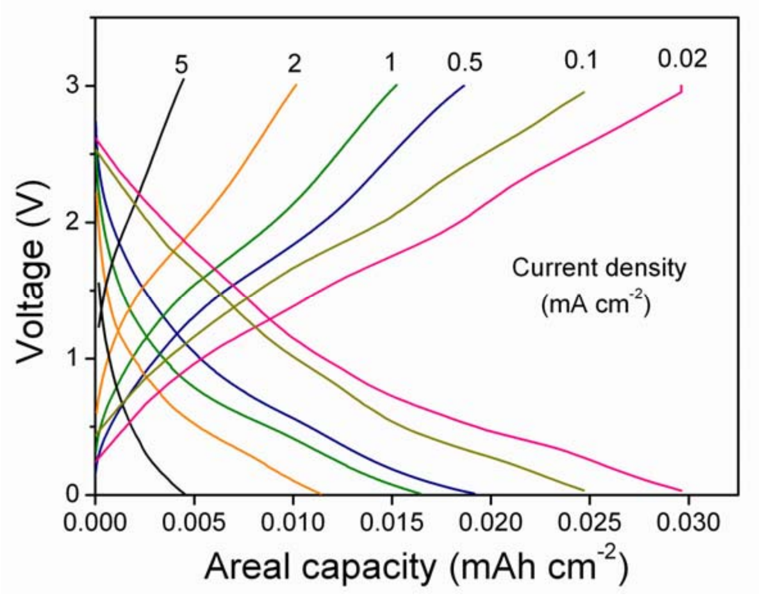

Supplementary Figure S16. Charge/discharge profiles of $KMnO_4$ treated S/NP Cu electrode at various current densities.

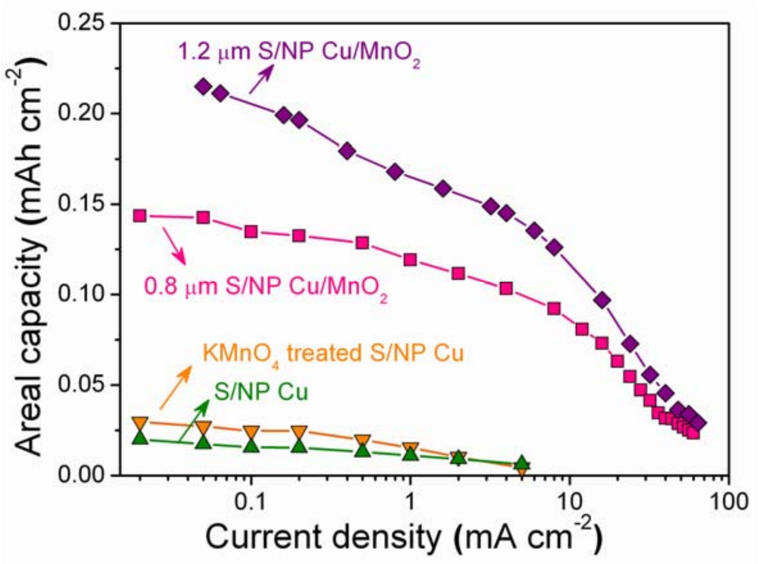

Supplementary Figure S17. Comparison of areal capacities at different current densities for S/NP Cu/MnO_2 with the 0.8- and 1.2-μm-thick NP Cu/MnO_2 layers, and bare and $KMnO_4$-treated S/NP Cu foils with 0.8-μm-thick NP Cu layers.

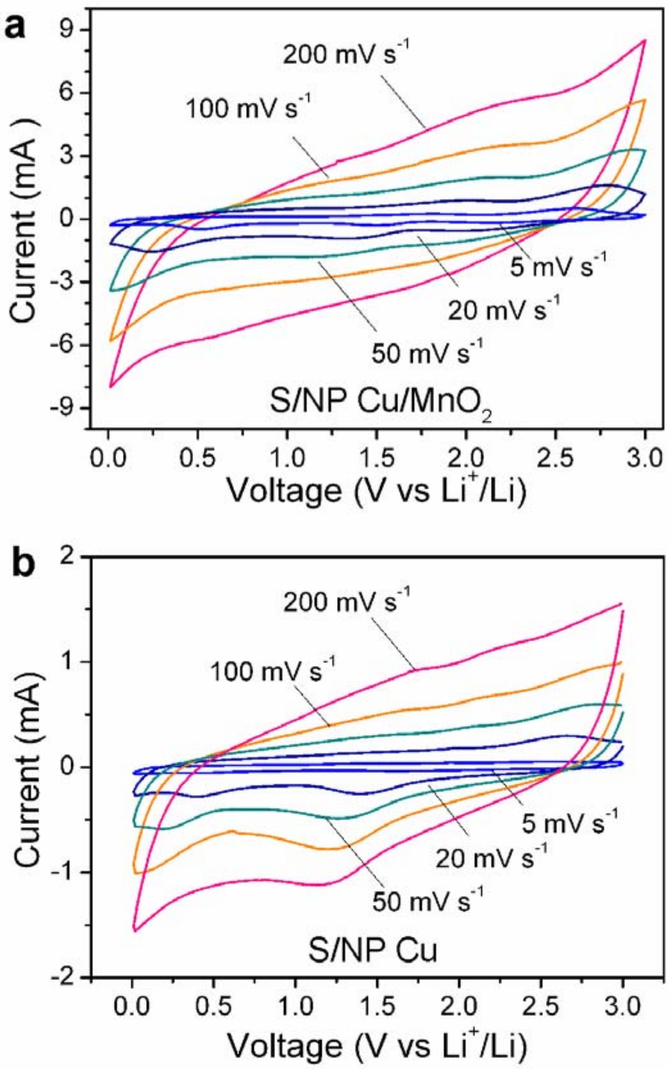

Supplementary Figure S18. Typical CV curves of Li ion batteries based on (a) S/NP Cu/MnO_2 hybrid and (b) S/NP Cu bulk electrode at different scan rates.

The power (P) and energy (W) densities of the constituent MnO_2 are calculated according to the CV curves obtained from Li-ion batteries of S/NP Cu/MnO_2 and S/NP Cu bulk electrodes in the light of the equations:

$$P_{\mathrm{MnO2}} = v(Q_{\mathrm{S/NP\ Cu/MnO2}} - Q_{\mathrm{S/NP\ Cu}})/m_{\mathrm{MnO2}}$$

$$E_{\mathrm{MnO2}} = \Delta V(Q_{\mathrm{S/NP\ Cu/MnO2}} - Q_{\mathrm{S/NP\ Cu}})/(3600m_{\mathrm{MnO2}})$$

Here $Q_{\mathrm{S/NP\ Cu/MnO2}} = (\int_{V_1}^{V_2} i(V)_{\mathrm{S/NP\ Cu/MnO2}}\mathrm{d}V)/v$, and $Q_{\mathrm{S/NP\ Cu}} = (\int_{V_1}^{V_2} i(V)_{\mathrm{S/NP\ Cu/}}\mathrm{d}V)/v$ are the charges of S/NP Cu/MnO_2 and S/NP Cu bulk electrodes, respectively, $\Delta V = V_2 - V_1$ with V_1 and V_2 being the two integration limits of cathode current $I(V)$, and m is

the mass of the constituent MnO_2, v denotes the scan rate.

The total capacity contributed from Li insertion and capacitive processes is calculated according to, $C_{Total} = \int_{V_1}^{V_2} i_{Total}(V)dV)/(3.6vm_{MnO2})$. While the insertion capacity is evaluated by, $C_{Insertion} = \int_{V_1}^{V_2} i_{Insertion}(V)dV)/(3.6vm_{MnO2})$, with $I_{Insertion}(V) = k_2(V)v^{1/2}$. The specific capacitance can be given by $C_{cap} = C_{Total}$-$C_{Insertion}$.

The power and energy densities of S/NP Cu/MnO_2 hybrid bulk electrode are calculated according to the equations:

$$P_{S/NP\ Cu/MnO2} = \int_{V_1}^{V_2} i(V)_{S/NP\ Cu/MnO2} dV/m_{S/NP\ Cu/MnO2},$$

$$E_{S/NP\ Cu/MnO2} = (\Delta V \int_{V_1}^{V_2} i(V)_{S/NP\ Cu/MnO2} dV)/(3600vm_{S/NP\ Cu/MnO2}).$$

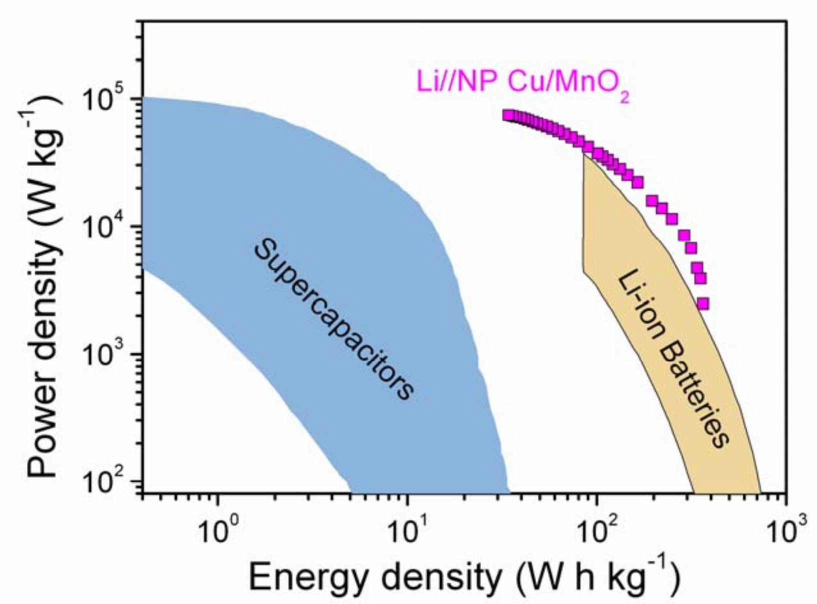

Supplementary Figure S19. The Ragone plot comparing the energy and power densities of the whole NP Cu/MnO_2 hybrid layer (the total mass of NP Cu/MnO_2 layer is included) to these of electrochemical supercapacitors, and lithium-ion batteries. The energy and power densities of electrochemical supercapacitor electrodes are estimated from the Ragone plot of the review paper [11] by multiplying a factor of 8. The distribution of energy and power densities of Li-ion battery electrodes refers to Ref. 14.

Supplementary references:

1. Fletcher, S., Barradas, R.G. & Porter, J.D. The anodic oxidation of copper amalgam and polycrystalline copper electrodes in LiOH solution. *J. Electrochem. Soc.* **125**, 1960-1968 (1978).
2. Yeo, B.S. & Bell A.T. Enhanced activity of gold-supported cobalt oxide for the electrochemical evolution of oxygen. *J. Am. Chem. Soc.* **133**, 5587-5593 (2011).
3. Trasatti, S. & Peterii O.A. Real surface area measurements in electrochemistry. *Pure & Appl. Chem.* **63**, 711-734 (1991).
4. Seker, E. *et al.* The effects of post-fabrication annealing on the mechanical properties of freestanding nanoporous gold structures. *Acta. Mater.* **55**, 4593-4602

(2007).

5. Serker, E. *et al.* The fabrication of low-impedance nanoporous gold multiple-electrode arrays for neural electrophysiology studies. *Nanotechnology* **21**, 125504 (2010).
